# Short term high-intensity interval training in patients scheduled for major abdominal surgery increases aerobic fitness

**DOI:** 10.1186/s13102-022-00454-w

**Published:** 2022-04-07

**Authors:** Anna Michel, Vincent Gremeaux, Guillaume Muff, Basile Pache, Sandrine Geinoz, Ana Larcinese, Charles Benaim, Bengt Kayser, Nicolas Demartines, Martin Hübner, David Martin, Cyril Besson

**Affiliations:** 1 Faculty of Biology and Medicine, University of Lausanne (UNIL), Lausanne, Switzerland; 2 Department of Sports Medicine, Swiss Olympic Medical Center, Lausanne University Hospital CHUV, University of Lausanne (UNIL), Lausanne, Switzerland; 3 Institute of Sport Sciences, University of Lausanne (UNIL), Lausanne, Switzerland; 4 Department of Physical Medicine and Rehabilitation, Lausanne University Hospital CHUV, University of Lausanne (UNIL), Lausanne, Switzerland; 5 Department of Visceral Surgery, Lausanne University Hospital CHUV, University of Lausanne (UNIL), Rue du Bugnon 46, 1011 Lausanne, Switzerland; 6 Department of Physiotherapy, Lausanne University Hospital CHUV, Lausanne, Switzerland

**Keywords:** Prehabilitation, CPET, Major abdominal surgery, Exercise, Aerobic capacity

## Abstract

**Background:**

Prehabilitation may improve postoperative clinical outcomes among patients undergoing major abdominal surgery. This study evaluated the potential effects of a high-intensity interval training (HIIT) program performed before major abdominal surgery on patients’ cardiorespiratory fitness and functional ability (secondary outcomes of pilot trial NCT02953119).

**Methods:**

Patients were included before surgery to engage in a low-volume HIIT program with 3 sessions per week for 3 weeks. Cardiopulmonary exercise and 6-min walk (6MWT) testing were performed pre- and post-prehabilitation.

**Results:**

Fourteen patients completed an average of 8.6 ± 2.2 (mean ± SD) sessions during a period of 27.9 ± 6.1 days. After the program, $\dot{\mathrm{V}}$O_2_ peak (+ 2.4 ml min^−1^ kg^−1^, 95% CI 0.8–3.9, *p* = 0.006), maximal aerobic power (+ 16.8 W, 95% CI 8.2–25.3, *p* = 0.001), $\dot{\mathrm{V}}$O_2_ at anaerobic threshold (+ 1.2 ml min^−1^ kg^−1^, 95%CI 0.4–2.1, *p* = 0.009) and power at anaerobic threshold (+ 12.4 W, 95%CI 4.8–20, *p* = 0.004) were improved. These changes were not accompanied by improved functional capacity (6MWT: + 2.6 m, 95% CI (− 19.6) to 24.8, *p* = 0.800).

**Conclusion:**

A short low-volume HIIT program increases cardiorespiratory fitness but not walking capacity in patients scheduled for major abdominal surgery. These results need to be confirmed by larger studies.

**Supplementary Information:**

The online version contains supplementary material available at 10.1186/s13102-022-00454-w.

## Background

Postoperative complications after major abdominal surgery are of public health and economic concern [1, 2]. The concept of Enhanced Recovery After Surgery (ERAS) contributes to reducing postoperative complications and improving patient comfort by implementing multimodal measures, starting in the preoperative period [3, 4]. Several surgical specialties now have started implementing training protocols in the preoperative period [5–8]. Prehabilitation, as a principle of preoperative training, for example through adapted physical activity, may improve the general condition of patients prior to surgery [9]. Preoperative training protocols, as well as their results, vary widely between studies. For abdominal surgery, several reviews summarized the effects of chest physiotherapy, strength and/or endurance training, or multimodal programs that combine exercise, and nutritional and psychological support, showing promising results [10–13]. Among exercise modalities, short high-intensity interval training (HIIT) (e.g. 15-s periods of intense exercise interspersed with 15-s recoveries) is considered a safe, time-efficient and effective mean to improve cardiorespiratory fitness in clinical populations [14–17]. Based on the protocols used in the initiation phase of cardiac rehabilitation patients with low exercise capacity, low-volume HIIT protocols appear to be more optimal [18]. These low-volume programs, performed at 80% of maximal aerobic power (MAP), are also already widely used and proved effective in patients with metabolic syndrome [19, 20].

Identifying the population at risk for complications post-surgery can be done in different ways [9]. Maximum oxygen consumption ($\dot{\mathrm{V}}$O_2_peak) and oxygen consumption at anaerobic threshold ($\dot{\mathrm{V}}$O_2_AT), which are measures of cardiorespiratory fitness, can be evaluated by Cardiopulmonary Exercise Testing (CPET). These measures are good predictors of all-cause mortality and cardiovascular events, and can also be used to predict morbidity risk after abdominal surgery [21–23]. In patients scheduled for resection of benign or malignant colorectal disease, $\dot{\mathrm{V}}$O_2_peak correlated well with functional effort capacity (6-min walk test (6MWT)), suggesting that pre-operative training may also improve functional capacity [24].

Prehabilitation’s challenge is to improve the cardiorespiratory fitness of patients in a limited time frame, in order to positively impact the postoperative outcomes. In preparation of a controlled clinical trial, a prospective study of the effects of a 3-week HIIT prehabilitation program in patients scheduled for elective major abdominal surgery was designed [25]. The present article reports the results of this pilot study on the efficacy of the training modality on cardiorespiratory fitness and walking performance.

## Methods

### Study design

This article reports secondary outcomes of a prospective pilot study in preparation of a clinical trial in patients undergoing elective major abdominal surgery at the Lausanne University Hospital (CHUV) between May 2017 and January 2020 [25]. The ethics committee of the Canton de Vaud (#469/15) approved the study. The study was registered on www.clinicaltrials.gov registry (NCT02953119) and was conducted in accordance with ethical standards of the Helsinki declaration. Written informed consent was obtained from all participants to the study.

Patients were included at the preoperative consultation by the operating surgeons, according to inclusion and exclusion criteria (supplementary material). Each patient gave written informed consent to participate. There was no extra surgery delay from participation to the study. Patients were then addressed at the Sports and Exercise Medicine Department for medical clearance and CPET.

### Cardiopulmonary exercise testing

The clinical check-up upon inclusion included complete clinical history and examination, resting electrocardiogram (ECG), and the measurement of blood pressure, weight and height. Participants then completed a maximal CPET on an ergocycle (Corival CPET, Lode, Netherlands) to determine maximal aerobic power (MAP) and peak oxygen consumption ($\dot{\mathrm{V}}$O_2_peak, highest value of 20-s average [26]). After a 3 min rest, participants started 3 min of unloaded pedaling at 60 rpm (revolutions per minute). Power was increased with a ramp protocol of 10 to 25 W per min according to Wasserman’s equation for exercise workload increments [27]. Oxygen consumption ($\dot{\mathrm{V}}$O_2_), expired carbon dioxide ($\dot{\mathrm{V}}$CO_2_) and minute ventilation were measured using a Cortex Metalyzer 3B gas exchange analyzer (Cortex Biophysik GmbH, Leipzig, Germany), which was calibrated for flow and gas concentrations before every procedure according to manufacturer recommendations. Stress ECG was monitored with a Custo Cardio 200 (Custo Med GmbH, Ottobrunn, Germany). The following maximum criteria were checked for each test: voluntary exhaustion, plateauing of the $\dot{\mathrm{V}}$O_2_–Work rate relationship ($\dot{\mathrm{V}}$O_2_ increasing by less than 2 ml min^−1^ kg^−1^ following a power increment), peak heart rate (HR) within 10 beats·min^−1^ of the age-predicted maximum, and peak RER above 1.10. CPET was considered as maximal if patients stopped because of exhaustion and if at least one of the other maximum criteria was met. Data on $\dot{\mathrm{V}}$O_2_peak, $\dot{\mathrm{V}}$O_2_AT, MAP, relative MAP and peak heart rate (HR_peak_) were excluded from analysis if those criteria were not met.

Two experienced exercise physiologists blindly determined $\dot{\mathrm{V}}$O_2_ and power at the anaerobic threshold ($\dot{\mathrm{V}}$ O_2_AT and P AT) with established criteria [26]: (1) excess $\dot{\mathrm{V}}$CO_2_ relative to $\dot{\mathrm{V}}$O_2_ above the AT with the modified V-slope method; (2) identifying hyperventilation relative to oxygen; and (3) excluding hyperventilation relative to CO_2_ at the AT inflection point identified by criteria 1 and 2 [26]. If the difference between the physiologists was ≤ 3%, the results were averaged. When the difference was > 3%, they each analyzed the test again and discussed until consensus. A sport physician and a cardiologist systematically interpreted CPET and patients were excluded in case of abnormal response to exercise [26]. Heart rate was extracted at rest, anaerobic threshold and peak. CPET was performed pre- and post-prehabilitation, for every patient according to the same standard procedures [26]. On the first and last day of the program, functional capacity was measured using the 6MWT [24]. Main variables were maximal aerobic power (MAP), aerobic capacity ($\dot{\mathrm{V}}$O_2_peak, $\dot{\mathrm{V}}$O_2_AT), and functional capacity assessed by the 6MWT.

### High-intensity interval training

Patients performed the prehabilitation program supervised by physiotherapists until the day of surgery. The training protocol was based on a model used in a comparable study in lung cancer patients [5]. The work-out, based on the patients' individual CPET results, consisted of a 5-min warm-up at 50% of MAP, followed by 2 series of 10 min of 15 s of high-intensity intervals at 80% of MAP interspersed by 15 s at 35% of MAP (active pedaling), with a 4-min break of unloaded pedaling in between. The work-out ended with a 5-min cool-down at 30% of MAP. This represented a short interval low-volume protocol, as previously described [19]. Finally, stretching exercises were performed with the patient: stretching of the sural triceps, the quadriceps, the hamstrings and the back. No resistance training exercises were performed. Session duration was approximatively 1 h and was adapted to each patient according to the results of their CPET. This training was performed 3 times a week for 3 weeks before surgery.

### Statistical analyses

Normality was assessed with Kolmogorov–Smirnov test. If normality passed, parametric statistics were used, and data are presented as mean, standard deviation (SD) and confidence interval (95% CI). When normality failed, non-parametric statistics were used, and data are presented as medians (interquartile range (IQR)). For comparisons, two-tailed paired t-tests were used for normally distributed data and Wilcoxon sign-rank tests for non-normal distributions. Statistical significance was set at *p* < 0.05. Correlations were assessed using Pearson correlation. GraphPad Prism, version 9.0.0 (86) and Excel, version 16.16.2 (180910) were used for analysis. Missing data were omitted based on the available case analysis (pairwise).

## Results

### Patients

Participation in the study was proposed to 44 patients fulfilling the inclusion criteria. Twenty of them agreed to participate, 3 were excluded for clinical reasons (abnormal CPET) and were referred for cardiology follow-up, and 1 patient had his surgery earlier. Two participants abandoned, finding the program too hard for their condition and lacking motivation. Fourteen patients completed the HIIT program. Their mean age was 64 ± 13.9 years with 8 male and 6 female patients. The mean BMI (Body Mass Index) was 28.4 ± 5.9 kg m^−2^. The mean number of completed training sessions was 8.6 ± 2.2 over a period of 27.9 ± 6.1 days between CPETs. Full adherence to the training program was 88% (i.e. no missed training sessions). After prehabilitation, one patient did not achieve maximal effort on the second CPET due to fatigue, dyspnea and mask discomfort. Another took off his mask during the second CPET due to discomfort and his $\dot{\mathrm{V}}$O_2_peak values are missing. There were no adverse events observed during prehabilitation.

### Effects of prehabilitation

**Fig. 1:**
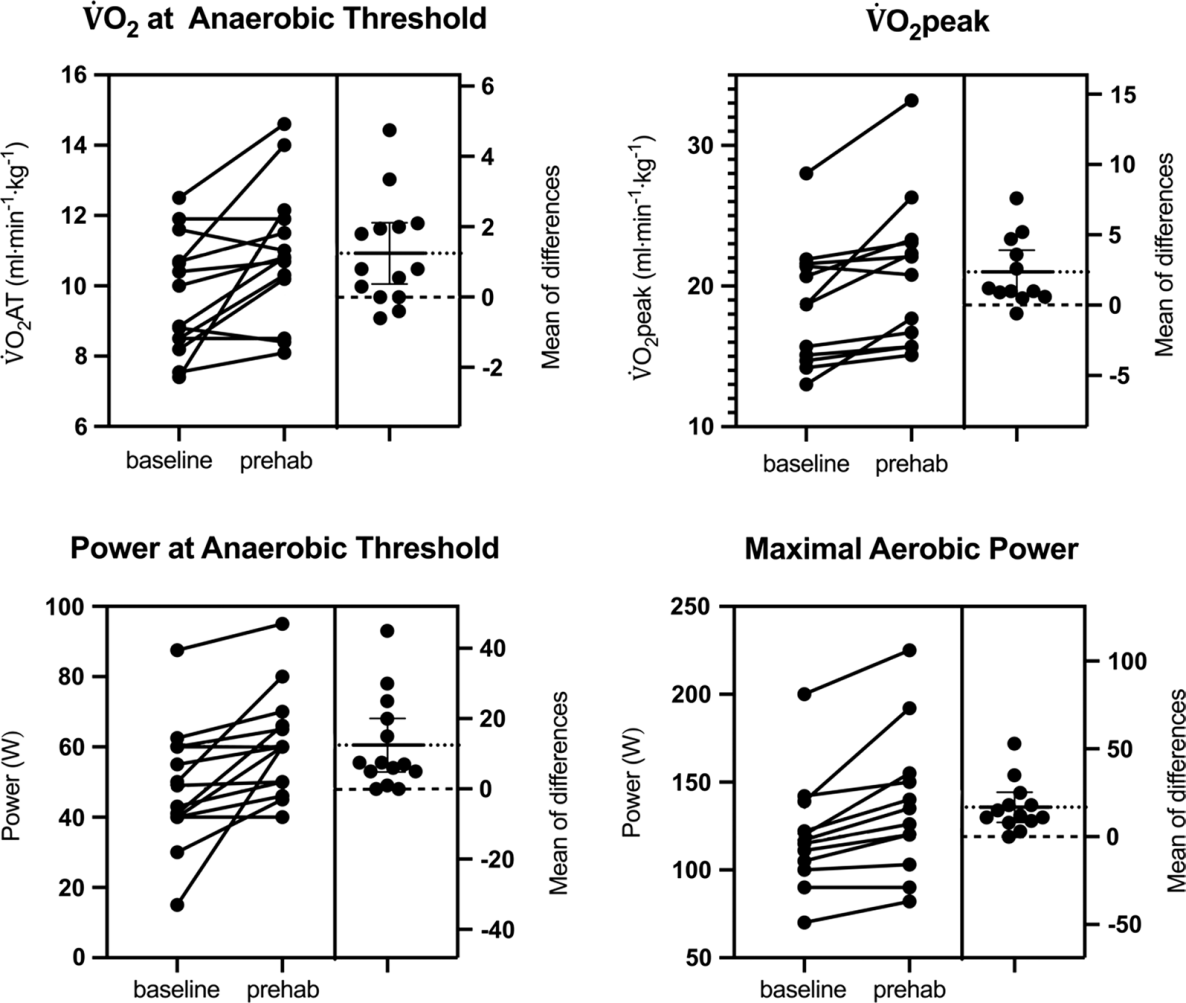
Individual responses to prehabilitation: aerobic capacities. $\dot{\mathrm{V}}$O_2_AT: oxygen uptake at anaerobic threshold (ml min^−1^ kg^−1^), $\dot{\mathrm{V}}$O_2_peak: maximal oxygen uptake (ml min^−1^ kg^−1^), Power at anaerobic threshold (P AT, watts), Maximal aerobic power (MAP, watts). Dotted line represents the mean of the differences between before and after prehabilitation. Dashed line represents the zero of the mean of differences

**Fig. 2:**
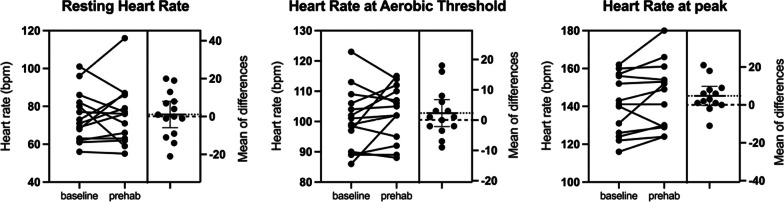
Individual responses to prehabilitation: heart rate. Bpm, beats per minute. Dotted line represents the mean of the differences between before and after prehabilitation. Dashed line represents the zero of the mean of differences

**Fig. 3:**
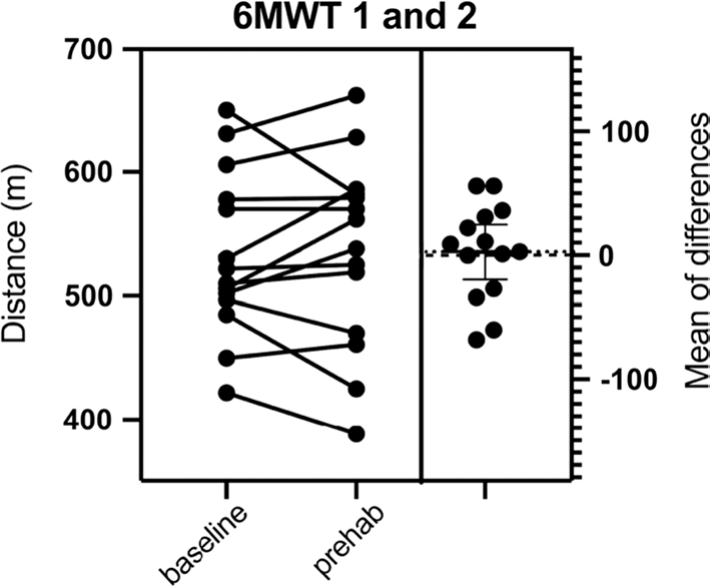
Individual responses to prehabilitation: functional testing. 6MWT 1: 6-min walk test (meters) at baseline, 6MWT 2: 6-min walk test (meters) after prehabilitation. Dotted line represents the mean of the differences between before and after prehabilitation. Dashed line represents the zero of the mean of differences

There were significant improvements in aerobic capacity and power output after prehabilitation, but not in functional testing and heart rate measures. Descriptive analyses of CPET results before and after the prehabilitation are shown in Table 1. Individual responses and absolute means of differences during CPET and 6MWT are shown in Figs. 1, 2 and 3. Patients had a significant increase in $\dot{\mathrm{V}}$O_2_peak of 13% (mean difference 2.4 ml min^−1^ kg^−1^, 95% CI 0.8–3.9, *p* = 0.006) as well as in $\dot{\mathrm{V}}$O_2_AT of 13% (mean difference 1.2 ml min^−1^ kg^−1^, 95%CI 0.4–2.1, *p* = 0.009).

**Table 1:** Descriptive analysis of cardiopulmonary exercise test results

	Baseline (n = 14)	Prehabilitation (n = 14)	*P* value
HR_rest_ (bpm)	74.6 (13.3)	75.7 (15.6)	0.745
HR_AT_ (bpm)	100.5 (10.3)	102.8 (8.9)	0.281
HR_peak_ (bpm)	140.8 (15.8)	145.5 (17.7)	0.060
$\dot{\mathrm{V}}$O_2_AT (ml min^−1^ kg^−1^)	9.7 (1.6)	10.9 (1.9)	0.009
$\dot{\mathrm{V}}$O_2_AT (l min^−1^) median, IQR	0.73 (0.2)	0.76 (0.2)	0.004
$\dot{\mathrm{V}}$O_2_ peak (ml min^−1^ kg^−1^)	18.6 (4.3)	21 (5.3)	0.006
$\dot{\mathrm{V}}$O_2_ peak (l min^−1^)	1.47 (0.5)	1.66 (0.6)	0.007
P AT (W)	48.1 (18.1)	65.5 (14.7)	0.004
MAP (W)	118.9 (30.8)	135.7 (39.2)	0.001
MAP relative (W kg^−1^) median, IQR	1.46 (0.4)	1.67 (0.5)	< 0.001
METS max	5.5 (1.2)	6.3 (1.4)	0.006

Data are presented in mean (SD). N = 14, unless clarified

CPET: cardiopulmonary exercise testing; HR_rest_: heart rate before CPET (beats per minute); HR_AT_: heart rate at anaerobic threshold (beats per minute); HR_peak_: heart rate at maximal oxygen uptake (beats per minute) (n = 13), $\dot{\mathrm{V}}$O_2_peak (ml min^−1^ kg^−1^): relative maximal oxygen uptake (n = 12, a patient took off his mask due to discomfort),$\dot{\mathrm{V}}$O_2_peak (l⋅min^−1^): absolute maximal oxygen uptake (n = 12, a patient took off his mask due to discomfort); $\dot{\mathrm{V}}$O_2_AT (ml min^−1^ kg^−1^): relative oxygen uptake at anaerobic threshold, $\dot{\mathrm{V}}$O_2_AT (l min^−1^): absolute oxygen uptake at anaerobic threshold; PAT: power at anaerobic threshold (W); MAP: maximal aerobic power (W) (n = 13); MAP relative: maximal aerobic power according to weight (W kg^−1^) (n = 13); METS: metabolic equivalent of task

Power at AT was significantly increased by 26% (mean difference 12.4 W, 95%CI 4.8–20, *p* = 0.004). MAP increased by 14% (mean difference 16.8 W, 95%CI 8.2–25.3, *p* = 0.001). The maximal relative aerobic power (MAP relative to body mass) increase was also significant (median difference 0.2 W kg^−1^, 97.95% CI 0.09–0.2, *p* = 0.007) (Fig. 1).

Heart rate at rest, at anaerobic threshold and at peak increased by 1.1 bpm (mean difference, 95%CI (− 5.9) to 8.0, *p* = 0.745), 2.3 bpm (mean difference, 95%CI (− 2.1) to 6.8, *p* = 0.281) and 4.8 bpm (mean difference, 95%CI (− 0.2) to 9.8, *p* = 0.060) respectively, but none of these differences reached statistical significance (Fig. 2).

**Fig. 4:**
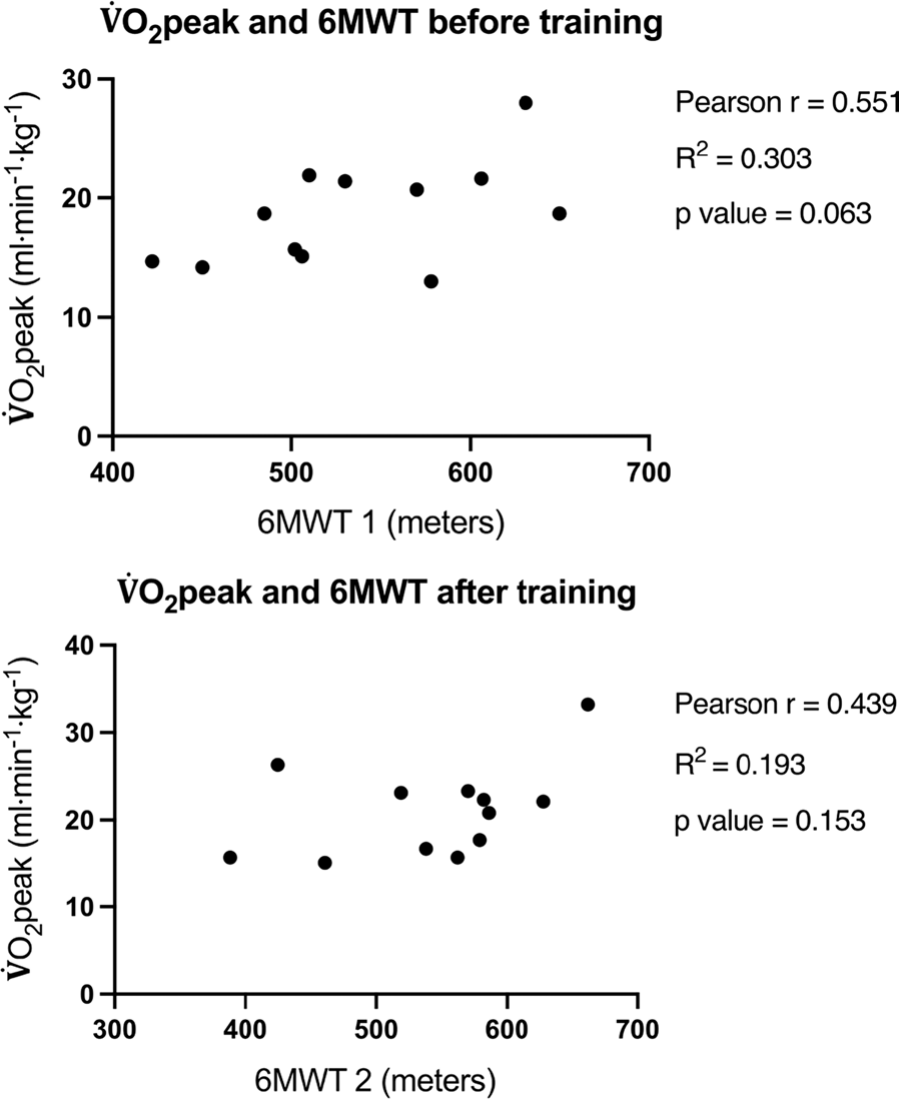
Correlation between maximum oxygen uptake and functional capacity before and after prehabilitation. 6MWT: 6-min walk test, $\dot{\mathrm{V}}$O_2_peak: maximal oxygen uptake (ml·min^−1^ kg^−1^)

There was no significant difference between the first 6MWT (baseline) and the second 6MWT (after prehabilitation, before surgery) (mean 539 m ± 70 vs. 542 m ± 76; mean difference + 3 m, 95% CI (− 20) to 25, *p* = 0.806) (Fig. 3). A correlation between the baseline $\dot{\mathrm{V}}$ O_2_peak and the walking distance measured (6MWT 1) before prehabilitation was found but was not significant (r = 0.55, 95% CI (− 0.03) to 0.9, R squared = 0.303, *p* = 0.063), see Fig. 4. After prehabilitation (6MWT 2), the correlation was less and not significant either (r = 0.44, 95% CI (− 0.2) to 0.8, R squared = 0.193, *p* = 0.153).

## Discussion

The main findings of the present study on the effects of a 3-week HIIT prehabilitation program in patients scheduled for major abdominal surgery were an increase in maximal and submaximal aerobic capacities, without any adverse effects. It thus proved possible to safely enhance exercise capacity of surgical patients scheduled for major abdominal surgery with a short-interval HIIT program over a limited time period.

Prehabilitation may enhance fitness levels and prepare patients to better cope with the stress caused by surgery [28]. The concept is to increase cardiorespiratory fitness and enhance functional reserve (i.e. a patient’s functional capacities engaged in case of effort or disease-caused stress [29]), and thus decrease pre- and postoperative complications [30]. While there are encouraging results for several types of scheduled surgery, there still is limited literature reporting the efficacy of short-term training programs aiming at increasing fitness levels in patients prior to major abdominal surgery. There are various forms and volumes of HIIT, and this could represent a key variable of how successful a prehabilitation program may be. We therefore tested the hypotheses that in such patients, a low-volume HIIT program enhances aerobic capacity ($\dot{\mathrm{V}}$O_2_peak and $\dot{\mathrm{V}}$O_2_AT) and maximal aerobic power (MAP), and secondarily, improves their functional performance as estimated by a walking test (6MWT). The program had to be effective and time-efficient (e.g. meaningful gains in a short period) as many surgical acts cannot be delayed.

### Improvement in physiological parameters

The significant increases in both $\dot{\mathrm{V}}$O_2_AT and $\dot{\mathrm{V}}$O_2_peak indicate a positive training response in these patients. These findings align with previous studies, despite the use of different interval protocols, either in volume and/or intensity [31]. Recent studies on patients scheduled for major abdominal surgery, liver resection of colorectal liver metastasis, or lung cancer patients, found that after about 4 weeks of HIIT, $\dot{\mathrm{V}}$O_2_peak had increased similarly, between 2 and 3 ml min^−1^ kg^−1^ [5, 9, 32], or led to an improvement in cycling endurance at 80% of peak aerobic power [33]. In comparison, a recent review on gastrointestinal and thoracic surgery also reported significant increases in $\dot{\mathrm{V}}$O_2_peak, up to 2.8 ml min^−1^ kg^−1^, using continuous exercise programs for at least 4 weeks [34]. HIIT may be more, or at least equally efficient, in terms of improvement in aerobic capacity among deconditioned patients who might have more difficulties maintaining longer efforts, and may be perceived as less difficult, compared to moderate continuous exercise programs [14–16]. Short-interval HIIT seems to be one of the most effective and safe training modalities, making it particularly suitable for the type of patients in the present study [5, 15, 17, 18, 35]. A recent review on cardiac rehabilitation for older patients with low functional capacity recommended to begin with short intervals, to then progress to medium- and long-interval HIIT, to amplify the accumulation of benefits from each protocol and thereby further increasing exercise and stress tolerance [16]. The present results' magnitude, 2.4 ml min^−1^ kg^−1^ increase in $\dot{\mathrm{V}}$O_2_peak, seems clinically relevant, as a 2 ml min^−1^ kg^−1^ is suggested by another on-going major abdominal surgery rehabilitation randomized controlled trial [36], and the finding that a 6% increase reduces time to all-cause mortality in chronic heart failure [37]. With regard to submaximal exercise, the clinical impact of an improved $\dot{\mathrm{V}}$O_2_AT was shown to be relevant from 1.5 ml min^−1^ kg^−1^ [32]. The present protocol elicited a significant 1.2 ml min^−1^ kg^−1^ improvement, slightly lower, suggesting room for improvement.

Along with enhancing aerobic capacities, maximal aerobic power (MAP) and power at anaerobic threshold also increased significantly. Bhatia et al. reported a significant increase in peak power output, along with an increase of HR_peak_, suggesting that part of the observed increase in aerobic capacity may have come from their patients having pushed themselves further after completing a HIIT program [5]. In the present results, HR_peak_ showed a tendency to increase, as shown in Fig. 2. An average 3.5% increase in HR_peak_ was observed during the second CPET compared to the first. It is, therefore, possible that a part of the 13% increase observed in aerobic capacity was due to patients being able to push themselves somewhat further even though CPET maximality was reached according to the criteria used.

### Functional data

Previous studies have supported that a small distance accomplished during 6MWT is a strong predictor of postoperative morbidity and, according to Lee et al., provides an alternative to CPET if not available [24]. That study also reported a positive correlation between walking distance and $\dot{\mathrm{V}}$O_2_peak (R^2^ = 0.52, *p* < 0.001). Despite the small number of participants in the present study, a similar trend between walking distance and $\dot{\mathrm{V}}$O_2_peak before training could be observed, although not reaching significance (Fig. 4). It is possible that increasing walking distance preoperatively with specific training could further contribute to reducing postoperative morbidity risk. However, direct impact of prehabilitation on postoperative complications remains debated with discordant results [12, 33, 38]. Bhatia et al. [5], using a similar training protocol as ours, showed a median increase of 20% (14–26%) of 6-min walking distance in patients with lung cancer and suggested that patients translated their increased aerobic power into better exercise capacities in daily life settings. Apart from following a HIIT program their patients were also actively encouraged to walk and carried a pedometer. Our protocol did not include any walking exercises, which potentially could explain why we did not find any significant improvement in 6MWT distance.

### Aerobic capacity and postoperative outcomes

Previous studies found that $\dot{\mathrm{V}}$O_2_peak and $\dot{\mathrm{V}}$O_2_AT are not only prime predictors for cardiovascular events and all-cause mortality [21, 23, 26], but also of morbidity of rectal cancer [22]. Prehabilitation prior to surgery favorable impacts on overall postoperative morbidity and pulmonary morbidity [6, 9, 10, 38]. A meta-analysis concluded that an increase of $\dot{\mathrm{V}}$O_2_peak by 3.5 ml min^−1^ kg^−1^ equals a 13–15% lower risk of all-cause mortality and cardiovascular mortality [21, 39]. Optimal CPET cut-offs to discriminate patients with potential greater postoperative morbidity were identified as < 18.6 ml·min^−1^·kg^−1^ of $\dot{\mathrm{V}}$O_2_peak and < 10.6 ml·min^−1^·kg^−1^ of $\dot{\mathrm{V}}$O_2_AT [22]. Depending on the type of pathology, those cut-offs may vary [23]. Along such cut-offs the patients of the present study could be considered low cardiorespiratory fit and at increased risk at baseline. Attesting to the relevance of training modalities such as used in the present study, the included patients who completed the program increased their $\dot{\mathrm{V}}$O_2_ above these cut-offs, underlining the potential clinical relevance of such programs.

### Limitations

Several limitations should be taken into account considering the results of this study. First, the number of included participants who finished the HIIT program was low. Second, in this pilot phase in preparation of a controlled trial, there was no control group receiving standard care but no training. Third, the training modality was based on stationary cycling and did not include any other types of exercise such as walking. Future studies could propose multi-modality programs complemented with general life-style advice and counseling [12, 13, 28]. Fourth, the subjective experience of the patients and any musculoskeletal issues for the 6MWT were not evaluated. Grading of rate of perceived exertion and dyspnea during HIIT should be included in future studies to study their evolution along the training sessions over time. Fifth, the age distribution of the present population, with 10 out of 14 patients over 65 years old, limits any conclusions to the effects of HIIT on the age-related fitness physiological decline [33]. Considering that the incidence and mortality rate of cancer is increasing in younger patients [40], prehabilitation may also be beneficial for younger patients as well, but remains to be studied specifically.

## Conclusion

A short (3 week) low-volume HIIT program before major elective abdominal surgery is safe and enhances aerobic capacities of patients with low baseline fitness but does not improve functional ability as quantified with the six-minute walking test. These results need to be confirmed by larger studies, and such programs need to be integrated more systematically in treatment plans.

### Practical implications

- A single modality preoperative stationary cycling HIIT program of 3 weeks before major elective abdominal surgery is safe and improves aerobic capacity.
- Such a short HIIT program does not confer improved functional ability as quantified with the 6-min walking test.

## Supplementary Information

**Additional file 1. **Inclusion and exclusion criteria.

## Data Availability

The datasets generated and/or analysed during the current study are not publicly available because these data are protected by the institution (CHUV), under cover of an ethical protocol (CER-VD), but are available from the corresponding author on reasonable request. The results of the feasibility of the program were presented in another article [25].
