# Supplementary material for: Short term high-intensity interval training in patients scheduled for major abdominal surgery increases aerobic fitness
Source: BMC Sports Sci Med Rehabil. 2022 Apr 7;14:61. doi: 10.1186/s13102-022-00454-w (PMC8991597; doi:10.1186/s13102-022-00454-w)
Supplement: Supplementary file 1 — Additional file 1. Inclusion and exclusion criteria. [file 13102_2022_454_MOESM1_ESM.docx]

**Additional File**

| **Supplementary material.** Inclusion and exclusion criteria. | |
| --- | --- |
| **Inclusion criteria** | - Any patient scheduled for elective major abdominal surgery, i.e. any resection of the esophagus, stomach, liver, pancreas, small intestine, colon or rectum for benign or malignant disease, as well as any other open or laparoscopic intra-abdominal procedure lasting more than 2 hours. - Scheduled surgery date > 4 weeks (allowing 3 weeks of training) |
| **Exclusion criteria** | - Patient under 18 years old. - Coronary artery disease defined by a stage ≥ III according to the Canadian Cardiovascular Society (1). - Heart disease defined by a stage ≥ III according to the New York Heart Association (2). - Uncontrolled cardiac arrhythmias. - Chronic Obstructive Pulmonary Disease defined by GOLD stage ≥ III (3). - Physical inability to ride a bike. - Orthopedic intervention in the last 6 weeks that does not allow resumption of physical activity. - Metabolic surgery and organ transplantation. |
| GOLD: Global Initiative for Chronic Obstructive Lung Disease | |

**References**

1. Campeau L. Letter: Grading of angina pectoris. Circulation. 1976 Sep;54(3):522–3.

2. Dolgin M, New York Heart Association, editors. Nomenclature and criteria for diagnosis of diseases of the heart and great vessels. 9. ed., 3. print. Boston, Mass.: Little, Brown and Co; 1994. 334 p. (A Little, Brown handbook).

3. Vestbo J, Hurd SS, Agustí AG, Jones PW, Vogelmeier C, Anzueto A, et al. Global Strategy for the Diagnosis, Management, and Prevention of Chronic Obstructive Pulmonary Disease: GOLD Executive Summary. Am J Respir Crit Care Med. 2013 Feb 15;187(4):347–65.
